# Encoding of Emotional Valence in Wild Boar (*Sus scrofa*) Calls

**DOI:** 10.3390/ani8060085

**Published:** 2018-06-05

**Authors:** Anne-Laure Maigrot, Edna Hillmann, Elodie F. Briefer

**Affiliations:** 1 Division of Animal Welfare, Veterinary Public Health Institute, Vetsuisse Faculty, University of Bern, Länggassstrasse 120, 3012 Bern, Switzerland; 2 Institute of Agricultural Sciences, ETH Zürich, Universitätsstrasse 2, 8092 Zürich, Switzerland; edna.hillmann@hu-berlin.de; 3 Agroscope, Swiss National Stud Farm, Les longs prés, 1580 Avenches, Switzerland; 4 Animal Husbandry, Albrecht Daniel Thaer-Institut, Faculty of Life Sciences, Humboldt-Universität zu Berlin, Philippstrasse 13, 10115 Berlin, Germany

**Keywords:** acoustic analysis, emotions, vocalization, welfare indicators, evolution

## Abstract

**Simple Summary:**

Animal welfare today is assessed based on both the physical and mental health of animals. However, measuring animal mental health, which includes emotions (i.e., short-term positive or negative reactions to specific events), remains a challenge. Since animals are known to use vocalizations to communicate their emotions to their peers, knowledge about how the structure of vocalizations changes with emotions could be very useful in order to develop noninvasive indicators for assessing animal welfare under captive conditions. The aim of this study was thus to investigate if the type of calls (i.e., grunt, scream, or squeal) or the acoustic structure of the calls emitted by captive wild boars changed according to the emotions they were experiencing. We found that wild boars used different types of calls in positive and negative situations. We also found that their acoustic structure changed according to the emotions. Indeed, calls produced in positive situations were generally shorter and at lower frequencies than those produced in negative situations. It thus seems that wild boars express their emotional state through their vocalizations. Overall, our study gives us better knowledge about how the emotions of captive wild boars could be assessed, and how this compares to domestic pigs.

**Abstract:**

Measuring emotions in nonhuman mammals is challenging. As animals are not able to verbally report how they feel, we need to find reliable indicators to assess their emotional state. Emotions can be described using two key dimensions: valence (negative or positive) and arousal (bodily activation or excitation). In this study, we investigated vocal expression of emotional valence in wild boars (*Sus scrofa*). The animals were observed in three naturally occurring situations: anticipation of a food reward (positive), affiliative interactions (positive), and agonistic interactions (negative). Body movement was used as an indicator of emotional arousal to control for the effect of this dimension. We found that screams and squeals were mostly produced during negative situations, and grunts during positive situations. Additionally, the energy quartiles, duration, formants, and harmonicity indicated valence across call types and situations. The mean of the first and second formants also indicated valence, but varied according to the call type. Our results suggest that wild boars can vocally express their emotional states. Some of these indicators could allow us to identify the emotional valence that wild boars are experiencing during vocal production and thus inform us about their welfare.

## 1. Introduction

Emotions are defined as short-lived and intense affective reactions to specific events or stimuli important to the organism [1]. They serve a crucial function, as they guide behavioral decisions in response to these triggering events or stimuli (e.g., approach or avoidance), including responses to social partners. They can be characterized by two key dimensions: valence (negative or positive; e.g., sad versus relaxed) and arousal (bodily activation or excitation; e.g., calm versus excited) [2]. The arousal dimension can be regarded as the intensity of bipolar valence (“arousal as intensity” version [3]). Negative emotions are part of the defensive motivational system and trigger avoidance behavior toward stimuli that threaten fitness, while positive emotions are part of the appetitive motivational system and trigger approach behavior toward stimuli that enhance fitness [3,4].

Although the existence of emotions in animals is now widely accepted [4], ideal indicators (e.g., fast, reliable, and noninvasive) that could enable us to assess these affective states are still lacking. In particular, there is a clear need to discover indicators of valence, which could enable us to discriminate negative from positive situations of similar emotional arousal, in order to promote situations triggering positive emotions and enhance animal well-being (“positive welfare” [5]). In many nonhuman mammals, vocalizations are assumed to be a direct expression of underlying emotions [6], since nonhuman animals have relatively limited voluntary control over the structure of their vocalizations compared to humans. Acoustic expressions of emotion have been found across species [7], even in those that can control vocal production, such as humans [8,9]. In most studied species, a change in emotional valence is associated with a change in call type (e.g., change from laughing to crying in humans, or from nickers to squeals in horses, *Equus caballus*) [7,10]. The best studied example is the rat (*Rattus norvegicus*), in which 22 kHz ultrasonic vocalizations (USVs) are produced in negative situations, while 50 kHz USVs are produced in positive situations [11]. However, valence can also be communicated through acoustic variants of the same call type. For example, the range of the fundamental frequency (F0) of elephant (*Loxondota africana*) rumbles diminishes in situations from negative to positive valence [12], while the energy distribution of cat (*Felis catus*) meows increases [13]. In dog (*Canis familiaris)* barks, the duration of calls decreases and F0 rises from negative to positive valence [14]. This acoustic variation, which can occur between call types as well as within a given call type, might be perceived by conspecifics and modulate social interactions (retreat or approach [15,16]). Within- and/or between-call types of changes occurring with the valence experienced by the producer of the vocalizations might thus constitute ideal indicators of emotion, especially for wild species that cannot easily be approached.

Indicators of emotion might also have the advantage of being valid across species. This would allow us to use the same set of indicators to assess the emotions of any domestic or wild nonapproachable species, and to enable direct between-species comparisons of emotional reactions to certain stimuli. Vocal indicators of emotional valence and arousal that are shared between species, and particularly between closely related species, could occur if vocal expression of emotions, as suggested by Darwin [17], has been conserved throughout evolution. There is now good evidence that this is the case for vocal expression of emotional arousal, suggesting that some vocal indicators of arousal could be shared across species [7,18]. However, whether this also applies to vocal indicators of valence remains unknown, since these indicators have been investigated in only a few species, and rarely in closely related species (but see [19]). So far, it seems that across several species, calls associated with positive emotions tend to be shorter, with a lower and less variable fundamental frequency (F0), compared to those associated with negative emotions [7,20].

In this study, we investigated vocal indicators of emotional valence in wild boars, in order to compare those indicators to the ones found in other species and in closely related domestic pigs (*Sus scrofa domesticus*, [7,21]), and to identify indicators that are shared across species. Wild boars live in large groups, including subadult males and adult females with their offspring, and are relatively vocal [22]. According to some recent research, they produce four main types of calls: grunts, squeals, grunt-squeals, and trumpets [23]. Yet, it is not known whether these call types can also vary with the emotion experienced by the producer, and whether the resulting indicators of emotion are similar to those found in domestic pigs [21]. Wild boars are the principal genetic source of modern European domestic pig breeds, which were domesticated 9000 years ago, although there is evidence for a post-domestication gene flow from wild boars to pigs [24]. The main genetic difference between the two species is that wild boars have 36 chromosomes, while domestic pigs have 38 [25]. A comparison of vocal indicators of emotion between these two species could shed light on the impact of domestication on emotion expression, and highlight the potential of vocal indicators of valence to be applied across species [19].

## 2. Materials and Methods

### 2.1. Studied Animals

The observations and recordings of 19 wild boars (12 females and 7 males) took place between February and April 2014 in 4 wildlife parks in Switzerland (Tierpark Dählhölzli Bern, Parc d’acceuil Pierre Challandes, Wildpark Bruderhaus, and Wildnispark Zürich Langenberg). All animals were housed in groups (6 groups of 2 to 7 animals). Their enclosures were 90–150 m^2^ and included a shelter. One group was kept in a larger enclosure, including a forest patch where they could forage in the soil *ad libitum*. All adult individuals had been in their group for at least 1 year. Fourteen individuals were categorized as adults (animals older than 3 years) and 5 were categorized as young (animals younger than 1 year). The animals were taken care of by park employees or volunteers. They were fed 2 times per day (at 8:30 in the morning and at 3:30 in the afternoon) with corn and various leftovers.

### 2.2. Observations and Recordings

We observed each group during 3 h each day, for as many days as there were individuals in the group (e.g., we observed a group of 3 animals for 3 days). Half of the observations were conducted from 7:00 a.m. to 10:00 a.m. (i.e., around the morning feeding time) and the other half from 2:00 p.m. to 5:00 p.m. (i.e., around the afternoon feeding time). The parks’ policies did not allow us to manipulate the animals. Therefore, recording of individual animals was performed opportunistically during presumed positive and negative expressions of emotions in naturally occurring situations [12,19,26].

### 2.3. Emotional Valence of the Situations

Three different situations were observed: anticipation of a food reward (considered as positive), affiliative interactions (considered as positive), and agonistic interactions (considered as negative; Table 1).

Since established behavioral indicators of emotion in wild boars are lacking, we used knowledge of the function of emotions and of wild boar behavior to assess the valence of the recording situations [27,28]. Encounters with rewarding stimuli that enhance fitness and trigger approach behavior toward the reward lead to positive emotions. In contrast, encounters with punishing stimuli that threaten fitness result in avoidance behavior and negative emotions [4]. We thus attributed a positive valence to the anticipation for food and affiliative interactions (Table 1). Conversely, we attributed a negative valence to agonistic interactions (Table 1) [4].

### 2.4. Emotional Arousal of the Situations

The emotional arousal that the animals were experiencing during vocal production was evaluated using body movements. This parameter was used as a control factor in our statistical model. Body movements have been revealed to be good indicators of arousal across species [29], including domestic pigs [21], and might also affect vocal parameters through changes in breathing patterns.

### 2.5. Data Collected

We recorded the calls from outside of the enclosures (between 5 and 45 m from the animals) with a directional microphone (Sennheiser MKH 70) connected to a digital recorder (Marantz PMD 661 MK II). We identified each animal using individual characteristics (e.g., body size, tail size, coat color, sex). Recordings were then uploaded to a computer at a sampling rate of 44.1 kHz and saved in WAV format at 16-bit amplitude resolution. All the acoustic analyses were performed using Praat v.5.3.61 DSP Package [30]. Calls were visualized on spectrograms with the following settings: FFT method, window length = 0.01 s, time steps = 1000, frequency steps = 250, Gaussian window shape, dynamic range = 60 dB. Calls with high levels of background noise and/or saturation present (as visualized on the spectrogram) were not selected for acoustic analysis. In addition, the situations in which vocalizations were produced were filmed whenever possible (when the camera was oriented toward the individuals that were vocalizing), using a Canon Legria FS2000 camcorder.

### 2.6. Data Analysis

**Figure 1:**
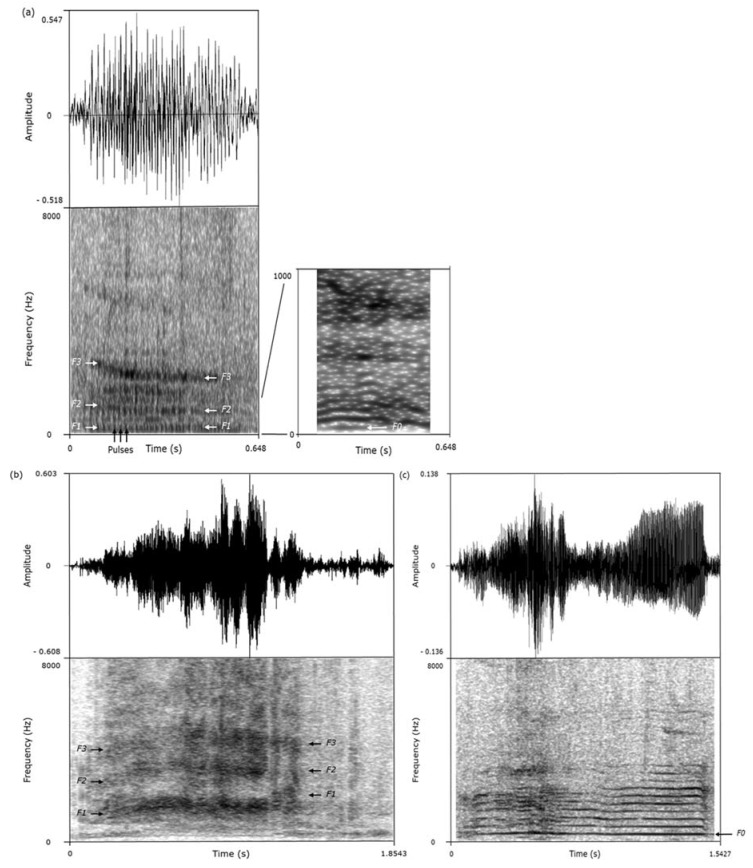
Oscillograms (above) and spectrograms (below) of (**a**) a grunt, (**b**) a scream, and (**c**) a squeal produced by wild boars. Fundamental frequency (F0) and F1, F2, and F3 (first three formants) are shown. These calls are available as audio files (Audio S1–S3).

We used the acoustic features of the calls to classify them as grunts, screams, or squeals (Figure 1, Table 2) [23,31]. This classification was performed while blind to the valence of the situations in which the calls had been recorded. As calls produced consecutively are more likely to be homogeneous, only the calls separated by at least 10 s intervals and of sufficient quality were considered for analysis (Table 3). 

The vocal parameters were extracted using a custom-built program in Praat [33], which batch-processed the analyses and the exporting of output data. To prevent biases related to the settings that we used for the analyses, the best settings to extract the vocal parameters of each individual were input in the script. Then, both negative and positive vocalizations of each animal were analyzed using the same settings. In total, we included 16 parameters in our analyses. The measured parameters are listed in Table 2 (further details can be found in the Supplementary Methods).

The videos of the recording situations were observed to score the body movements, using Interact software v. 9.0.7 (Mangold International GmbH, Arnstorf, Germany). Observations were made for 20 s, starting 10 s before each call. We then used the frequency of movement (proportion of time spent walking or running during these 20 s) as an indicator of emotional arousal for our analyses. The arousal scores were available for 154/256 calls, because it was not always possible to film every instance of vocal production.

### 2.7. Statistical Analysis

Linear mixed effects models (LMMs) were conducted in order to evaluate the effect of emotional valence on the vocal parameters. R (version 3.3.1, R Development Core Team, 2015) was used to perform the statistical analyses using the lmer function from the lme4 library [34]. The vocal parameters were used as response variables (one model per parameter). The fixed factors were the sex (female or male), age (young or adult), and number of individuals in the group (2 to 7) in order to control for the effects of the parameters. The factors of interest were the type of call (grunt, scream, or squeal), the valence (positive or negative), and the interaction between these two parameters. Finally, the random structure of the models was as follows: the situation nested within the identity of the animals, itself nested within their group, crossed with the date of the observations. This allowed us to control for repeated measurements of the same individuals, and for differences between groups and days. We removed any nonsignificant interactions from the models [35]. When an interaction was significant, we performed further post hoc tests by comparing the changes due to the emotional valence in each type of call separately using Tukey’s honest significant difference (HSD) test including the same control and fixed and random effects.

Because body movement (arousal score) was available for only 154/256 calls, we ran a second series of models on the reduced sample. To this aim, we used the same LMMs as for the first series of models described above, and added the body movements (proportion of time spent in movement, an indicator of arousal level) as an additional fixed factor.

The residuals of every model were checked graphically for normal distribution and homoscedasticity. We used a log transformation on *F0mean*, *AMextent*, *Q25*, *Q50*, *Q75*, *Duration*, *F0AbsSlope*, and *F2range* (see Table 2 for abbreviations) and a square root transformation on *Harmonicity* to satisfy these assumptions. The transformed parameters were then input into models fitted with a Gaussian family distribution and identity link function. We used the anova function of the lmerTest package in R to calculate *p*-values based on Satterthwaite’s approximations, and all the models were fitted with restricted maximum likelihood (REML) estimation. Additionally, we used Chi-square tests to compare the call types’ distribution across valences and situations to a set of expected values. We set the significance level at α = 0.05.

We present the results as the residuals of the models carried out, after controlling for all fixed factors with the exception of the factor of interest (e.g., without the factor “valence” when testing the difference between negative and positive situations, which corresponds to the response variable after removing the variance related to the control factors; raw values are available in Supplementary Tables S1–S3). All means are given with standard deviations (SDs).

### 2.8. Ethics

All observations of the animals were carried out in accordance with the “Guidelines for the treatment of animals in behavioural research and teaching” of the Association for the Study of Animal Behaviour (ASAB, 2012) and the current laws of Switzerland. The wildlife parks where the animals were studied are all open to the public, and the animals are well accustomed to the presence of surrounding visitors. This enabled us to be close enough to the animals to conduct observations from outside of the enclosures. Therefore, the animals were never manipulated during the experiment and all our data were collected by observation only.

## 3. Results

### 3.1. Proportion of Grunts, Squeals, and Screams

Squeals (χ-square: *χ*^2^_1_ = 26.56, *p* < 0.0001) and screams (χ-square: *χ*^2^_1_ = 38.35, *p* < 0.0001) were almost exclusively produced in negative situations (agonistic interactions) compared to positive ones (Table 3). Contrastingly, grunts were produced more in positive situations (anticipation for food, affiliative interactions) than in negative ones (χ-square: *χ*^2^_1_ = 7.71, *p* = 0.006; Table 3). Additionally, grunts appeared to be produced more often while anticipating a food reward than during affiliative interactions (χ-square: *χ*^2^_1_ = 12.57, *p* = 0.0004; Table 3). The number of screams and squeals produced while anticipating a food reward and during affiliative interactions did not differ significantly (*p* = 1 for all; Table 3).

### 3.2. Emotional Valence

We found 10 parameters that were significantly influenced by emotional valence. *Q25*, *Q50*, *Q75*, *Duration*, *F1range*, *F2mean*, *F2range*, *F3mean*, and *F3range* all decreased from negative to positive valence, while *Harmonicity* increased (Table 4 and Supplementary Table S1). The other parameters did not change significantly with the putative emotional valence (LMM: *p* ≥ 0.057 for all). 

After adding the proportion of movement (used as an indicator of emotional arousal) to those models, we found nine parameters that remained influenced by the valence of the emotion. *Q25*, *Q50*, *Q75*, *Duration*, *F2mean*, *F2range*, *F3mean*, and *F3range* all changed with valence in the same direction as they did without the proportion of movements (Table 5 and Supplementary Table S1). In addition, *AMrate* significantly decreased from negative to positive valence (Table 5 and Supplementary Table S1). To summarize, the calls produced in situations of positive valence were shorter, with less amplitude modulation (*AMrate*), lower frequencies (energy quartiles and formants), and a smaller range of the second and third formants.

### 3.3. Call Type

We found seven parameters that significantly differed between grunts, squeals, and screams. Two of the three energy quartile–related parameters (*Q25* and *Q50*) were lowest in grunts and highest in screams, while *Q75* and *Duration* were lowest in grunts and highest in squeals. The last two parameters (*MeanF0* and *F0AbsSlope*) could only be measured in grunts and squeals, and were lowest in grunts and highest in squeals (Table 6 and Supplementary Table S2). All other parameters did not significantly differ between call types (LMM: *p* ≥ 0.12 for all).

To summarize, squeals were the longest vocalizations and grunts the shortest. Screams had a higher energy distribution (*Q25* and *Q50*) than grunts and squeals, although squeals had a higher *Q75*. Squeals also had a higher fundamental frequency (*MeanF0*) and a steeper F0 slope (*F0AbsSlope*) than grunts.

### 3.4. Interaction between Valence and Call Type

The analysis of the interaction effect between emotional valence and call type revealed two parameters, *AMrate* and *F1mean*, for which call type influenced valence-related changes (LMM, interaction effect: *AMrate*, *F*_2,189_ = 3.45, *p* = 0.034; *F1mean*, *F*_1,143_ = 143.73, *p* < 0.0001). Post hoc tests showed that *AMrate* decreased from negative to positive valence in screams, while it did not vary significantly in either grunts or squeals (Table 7 and S3). On the other hand, *F1mean* decreased from negative to positive valence in grunts, while it increased in screams (Table 7 and S3).

Adding body movement (an indicator of emotional arousal) to each model revealed three parameters (*F1mean*, *F0mean*, and *Harmonicity*) that were influenced by the interaction between call type and valence (LMM, interaction effect: *F1mean*, *F*_1,66_ = 231.86, *p* < 0.0001; *F0mean*, *F*_1,92_ = 5.86, *p* = 0.017; *Harmonicity*, *F*_1,71_ = 4.85, *p* = 0.031). *F1mean* did vary in the same way as when body movement was not included (Table 5 and Supplementary Table S3). Additionally, from negative to positive valence, *F0mean* did not vary significantly in grunts, while it increased in screams (Table 5 and Supplementary Table S3). Finally, post hoc tests for each call type did not show any significant effect of emotional valence on *Harmonicity* for either grunts or squeals (Table 5 and Supplementary Table S3). All other parameters were not significantly affected by the interaction between type of call and emotional valence (*p* ≥ 0.066 for all). To summarize, the direction of changes linked to the valence of the recording situation varied depending on call type for the first formant (*F1mean*), the mean of the fundamental frequency (*F0mean*), and the degree of acoustic periodicity (*Harmonicity*).

## 4. Discussion

We tested whether the emotional valence of a situation affects the acoustic structure of wild boar calls. We found that grunts were produced more often in putatively positive situations (affiliative interactions and anticipation for a food reward), while squeals and screams were almost exclusively produced in putative negative situations (agonistic interactions). Within positive situations, grunts were produced especially when anticipating a food reward. In addition, for each call type, several parameters differed between negative and positive valence, independent of body movements (indicator of arousal and/or effect of breathing). These vocal indicators of valence might regulate social interactions and could help us to identify whether the animals experience positive or negative emotions. In particular, correlates of valence that are shared with other species could be very useful as cross-species indicators of emotion and welfare.

### 4.1. Valence 

We found variation in the types of vocalizations emitted according to the emotional valence attributed to the situation. Indeed, grunts were mainly produced in positive situations, while squeals and screams were almost entirely produced in negative situations. Additionally, after controlling for variations due to the proportion of body movement (indicator of emotional arousal and/or effect of breathing), we found that all parameters describing the second and third formants (*F2mean*, *F2range*, *F3mean*, and *F3range*) as well as the three energy quartiles (*Q25*, *Q50*, and *Q75*), the amplitude modulation rate (*AMrate*), and call duration dropped from negative to positive emotional valence. Formant-related parameters and distribution of energy are dependent on the shape and length of the vocal tract [36]. These parameters can be altered, notably by retraction of the larynx, as has been observed in goats (*Capra hircus*), dogs (*Canis familiaris*), domestic pigs, cotton-top tamarins (*Sagunius oedipus*) [37], and other species, such as fallow deer (*Dama dama*) [38]. The decrease that we observed in all these parameters could thus be explained by a stronger laryngeal retraction in positive situations. In primates, it has also been found that slight changes in the configuration of the nasal and oral cavities, such as mouth opening/closing, lip protrusion/retraction, and lip rounding/spreading, can have an impact on the formant’s frequencies [39,40,41]. Some of these changes (e.g., mouth opening/closing) could also explain the differences that we observed in the energy distribution and formant frequencies of wild boar calls. However, further analysis of the animals’ behavior while vocalizing would be necessary to find out the effect of these various changes on call structure. A similar decrease in the energy quartiles and/or formants from negative to positive emotions can be observed in Przewalski’s horses [19], domestic horses [27], and squirrel monkeys [42]. In addition, a decrease in duration from negative to positive situations seems to be common across species [7,20].

In addition to the above-mentioned changes, the mean of the first formant (*F1mean*) varied in a different way, with the valence as a function of call type in which this parameter was measured. Indeed, F1mean, which could only be measured in harsh and/or low-frequency calls (i.e., grunts and screams), increased from negative to positive valence in screams, while it decreased in grunts, independent of whether or not we controlled for emotional arousal (i.e., included the proportion of movement as a control factor in the model). One explanation for these differences could be that these call types have different functions, which also affect the call structure, leading to call-type-specific changes in parameters between emotions [43]. In domestic pigs, grunts are produced in a great variety of situations, while screams mainly indicate negative situations [44]. Therefore, grunts can be produced while eating, greeting conspecifics, being isolated, or anticipating (pleasant or unpleasant [45]), while screams are mainly produced during agonistic interactions (e.g., fighting, defending, showing aggression [31]). The same results (describing grunts as calls produced in diverse situations and squeals/screams produced mainly in agonistic interactions) have recently been found in wild boars [23]. In our study, grunts were observed in both positive and negative situations, while screams were produced mainly in negative ones. Indeed, only two screams (and four squeals) were recorded in the putative positive situations. These calls might have been produced by animals experiencing a negative emotion, although the situation was identified as positive according to our criteria (Table 1). Another explanation could be that the production mechanisms of these call types are different. Indeed, as explained above, it has been found in primates that formant frequencies can be influenced by changes in the configuration of the nasal and oral cavities [39,40,41]. In addition, Garcia et al. [46] suggested that in domestic pigs, some grunts might be nasally produced, while screams could be produced with the mouth open. These different production mechanisms could also affect the changes in sound structure that we found as a function of the valence of the situation. Overall, our results show that wild boars are able to express emotional valence through both the type of call they produce and acoustic changes within each call type.

### 4.2. Call Type

We found several differences in acoustic structure between wild boar call types. Compared to grunts, squeals and screams showed higher energy quartiles (*Q25*, *Q50*, and *Q75*) and were longer. In addition, the mean F0 (*F0mean*) and the F0 absolute slope (*F0AbsSlope*) were both higher in squeals than in grunts. These results are similar to those previously described for this species by Garcia et al. [23], who found that both duration and energy distribution were higher in squeals/screams than in grunts, and could be explained by the situation in which call types were produced. According to Morton’s “motivation-structural rule” [47], vocalizations produced in appeasing and fearful situations are generally high and tonal, while vocalizations produced in hostile situations tend to be low in frequency and harsh. August and Anderson [48] later added that friendly situations could be associated with soft, low-frequency rhythmic sounds, as has been described in carnivores (e.g., “purring” in cats [49]). The call structure of wild boars thus does partially comply with the motivation-structural rules, with high-frequency squeals (high in frequency and relatively pure tone) and screams (high in frequency and relatively harsh) being produced in hostile situations (agonistic interactions in our study, which are likely fearful in the case of the victim), and grunts (low in frequency) being observed mostly in friendly situations (e.g., affiliative interactions in our study; [47,48]). The same results have been found in Przewalski’s horses, which produce more whinnies and squeals (high-frequency calls) during agonistic interactions, and more nickers (low-frequency calls) during affiliative interactions [19].

### 4.3. Comparison with Domestic Pigs

In domestic pigs, positive grunts have been described as shorter, with a lower peak frequency and *F0* contour and a shorter range of *F3*, as well as a higher energy distribution (*Q25*–*75*) and formants (*F1*–*F3*), compared to negative grunts (Briefer et al., in prep.). Similarly, it has been found that pigs conditioned to expect a positive outcome produce calls with higher *Q25* and *Q50* compared to pigs conditioned to expect a negative outcome [21]. Some of these results are similar to what we found in wild boars. Indeed, in our study, *F3range* and *Duration* were also lower in positive than negative valence. By contrast, the energy distribution and frequencies of the formants were lower in positive than negative valence, while the opposite seemed to occur in domestic pigs. These differences suggest that evolution and/or the process of domestication had an influence on the vocal expression of emotions. It may be due to the reduction of predation pressure related to modern housing systems, or to differences in the range of emotions that they experience or express. Such differences in vocal expression of emotional valence have also been observed between the closely related domestic and Przewalski’s horses [19,27]. In those previous studies, we found that domestic horses communicate emotional valence mainly by using call duration and the average frequency of the highest fundamental frequency [27], while these parameters did not vary with valence at all in Przewalski’s horses [19]. This suggests that, unlike indicators of emotional arousal, indicators of valence have been altered during evolution or during the domestication process. Overall, only a limited number of parameters (*Duration* and *F3range*) could thus be used as indicators of emotional valence to assess welfare in both wild boars and domestic pigs.

## 5. Conclusions

We found some promising vocal indicators of emotional valence in wild boars. Indeed, the energy quartiles (*Q25*, *Q50*, and *Q75*), the duration, and the formants (*F1range*, *F2mean*, *F2range*, *F3mean*, and *F3range*) could all constitute good indicators of emotional valence across call types and situations. Some of these noninvasive indicators are similar to the ones found in domestic pigs (*Duration* and *F3range*), while others change in the opposite direction (energy distribution and formant frequencies). It thus seems that vocal expression of emotional valence has not been completely conserved throughout evolution and/or domestication.

## Figures and Tables

**Table 1:** Description of the recording situations, along with the associated behaviors and putative valence [22].

Situation	Description	Valence
Anticipation of a food reward	The animals could see the caretaker coming toward them with food (maximum 1 min)	Positive
Affiliative interactions	Interactions leading to decreased distance between the animals (approach behavior; e.g., allogrooming), in the absence of any aggressive behavior	Positive
	Allogrooming: an animal explores another’s skin with the nose and removes parasites and mud with the teeth	
Agonistic interactions	Interactions leading to an increased distance between animals (avoidance behavior; e.g., attack, chase)	Negative
	Attack: a wild boar physically attacks another by pushing or biting it	
	Chase: a wild boar pursues another	

**Table 2:** Abbreviations and definitions of the call types and vocal parameters [22,23,31,32].

Abbreviation	Measured in Which Call Type	Parameter Description
**Call types**		
Grunt		Pulsatile, low-frequency, and short-duration call
Scream		Long, harsh, and high-frequency call
Squeal		Long, loud, relatively tonal and high-frequency call
**Acoustic parameters**		
*F0mean* (Hz)	Grunt and Squeal	Mean F0 frequency value across the call
*F0AbsSlope* (Hz/s)	Grunt and Squeal	F0 mean absolute slope
*AMextent* (dB)	Grunt, Scream, and Squeal	Mean peak-to-peak variation of each amplitude modulation
*AMrate* (s–1)	Grunt, Scream, and Squeal	Number of complete cycles of amplitude modulation per second
*Q25* (Hz)	Grunt, Scream, and Squeal	Frequency value at the upper limit of the first quartile of energy
*Q50* (Hz)	Grunt, Scream, and Squeal	Frequency value at the upper limit of the second quartile of energy
*Q75* (Hz)	Grunt, Scream, and Squeal	Frequency value at the upper limit of the third quartile of energy
*Duration* (s)	Grunt, Scream, and Squeal	Total duration of the call
*Harmonicity*	Grunt and Squeal	Degree of acoustic periodicity (signal-to-noise ratio)
*F1mean* (Hz)	Grunt and Scream	Mean frequency value of the first formant
*F1range* (Hz)	Grunt and Scream	Difference between maximum frequency and minimum frequency of the first formant
*F2mean* (Hz)	Grunt and Scream	Mean frequency value of the second formant
*F2range* (Hz)	Grunt and Scream	Difference between maximum frequency and minimum frequency of the second formant
*F3mean* (Hz)	Grunt and Scream	Mean frequency value of the third formant
*F3range* (Hz)	Grunt and Scream	Difference between maximum frequency and minimum frequency of the third formant

**Table 3:** Number of grunts, screams, and squeals analyzed according to the valence and the situation for each individual (including range; *n* = 19 animals).

Valence	Situation	Grunt	Scream	Squeal
Negative	Agonistic interactions	58	44	37
Positive	Anticipation for food	63	1	2
	Affiliative interaction	29	1	2
Total positive calls		92	2	4
Total		150	46	41
Number per animal (mean ± SD)		7.9 ± 5.0	2.4 ± 2.3	2.2 ± 1.6
Range per animal		0 to 17	0 to 8	0 to 5

**Table 4:** Effect of valence attributed to the recording situations on the measured acoustic parameters (only significant values are shown). Residuals of the models controlled for the following factors: sex, age, size of the group, and type of call (see Supplementary Table S1 for raw values). The direction of the effect is displayed (< denotes an increase from negative to positive valence; > denotes a decrease).

Parameters	NumDF	DenDF	*F* Value	*Pr* (>*F*)	Valence	Mean	SD	Variation
*Q25*	1	229.53	17.72	<0.0001	Neg	0.062	0.473	>
					Pos	−0.088	0.282	
*Q50*	1	44.53	14.09	0.001	Neg	0.040	0.392	>
					Pos	−0.057	0.311	
*Q75*	1	223.90	8.53	0.004	Neg	0.042	0.402	>
					Pos	−0.060	0.364	
*Duration*	1	76.17	9.44	0.003	Neg	0.043	0.415	>
					Pos	−0.060	0.293	
*Harmonicity*	1	52.21	5.83	0.019	Neg	−0.053	0.533	<
					Pos	0.051	0.483	
*F1range*	1	55.61	8.56	0.005	Neg	10.631	101.185	>
					Pos	−11.694	60.329	
*F2mean*	1	60.58	11.14	0.001	Neg	31.598	173.624	>
					Pos	−35.149	217.512	
*F2range*	1	65.00	27.04	<0.0001	Neg	0.091	0.382	>
					Pos	−0.102	0.369	
*F3mean*	1	62.24	5.59	0.021	Neg	26.610	222.642	>
					Pos	−28.949	264.438	
*F3range*	1	183.87	49.91	<0.0001	Neg	0.094	0.385	>
					Pos	−0.102	0.329	

**Table 5:** Effect of valence attributed to the recording situations on the measured acoustic parameters (only significant values are shown). Residuals of the models controlled for the following factors: body movement, sex, age, size of the group, and type of call (see Supplementary Table S1 for raw values). The direction of the effect is displayed (< denotes an increase from negative to positive valence; > denotes a decrease).

Parameters	NumDF	DenDF	*F* Value	*Pr (>F)*	Valence	Mean	SD	Variation
*AMrate*	1	110.00	9.81	0.002	Neg	0.19	1.68	>
					Pos	−0.72	1.59	
*Q25*	1	128.57	7.69	0.006	Neg	0.05	0.45	>
					Pos	−0.13	0.34	
*Q50*	1	127.13	6.58	0.011	Neg	0.03	0.38	>
					Pos	−0.08	0.37	
*Q75*	1	129.26	4.13	<0.0001	Neg	0.03	0.38	>
					Pos	−0.09	0.41	
*Duration*	1	22.34	12.25	0.002	Neg	0.05	0.41	>
					Pos	−0.13	0.26	
*F2mean*	1	27.44	7.61	<0.0001	Neg	25.06	173.88	>
					Pos	−60.15	240.36	
*F2range*	1	26.54	32.21	<0.0001	Neg	0.07	0.38	>
					Pos	−0.17	0.31	
*F3mean*	1	33.08	5.02	<0.0001	Neg	23.36	231.58	>
					Pos	−52.55	267.14	
*F3range*	1	88.63	19.80	<0.0001	Neg	22.95	135.09	>
					Pos	−51.63	60.94	

**Table 6:** Effect of call type on the measured acoustic parameters (only significant values are shown). Residuals of the models controlled for the following factors: sex, age, size of the group, and valence (see Supplementary Table S2 for raw values). The variation between calls is displayed as follows: + denotes the highest value; − denotes the lowest.

Parameters	NumDF	DenDF	*F* Value	*Pr (>F)*	Call Type	Mean	SD	Variation
*F0mean*	1	183.39	449.50	<0.0001	Grunt	−0.163	0.264	−
					Squeal	0.598	0.447	+
*F0AbsSlope*	1	181.17	89.41	<0.0001	Grunt	−0.145	0.470	–
					Squeal	0.531	0.634	+
*Q25*	2	222.08	52.08	<0.0001	Grunt	−0.164	0.363	−
					Scream	0.343	0.470	+
					Squeal	0.215	0.684	
*Q50*	2	216.41	44.77	<0.0001	Grunt	−0.143	0.374	−
					Scream	0.283	0.464	+
					Squeal	0.205	0.539	
*Q75*	2	225.11	11.83	<0.0001	Grunt	−0.076	0.376	−
					Scream	0.122	0.437	
					Squeal	0.142	0.471	+
*Duration*	2	224.68	29.35	<0.0001	Grunt	−0.103	0.325	−
					Scream	0.159	0.437	
					Squeal	0.193	0.437	+

**Table 7:** Effect of the interaction between the emotional valence of the recording situations and call type (only significant values are shown); residuals of the models with and without body movement as a control factor. The models were controlled for the following factors: sex, age, size of the group, and valence (see Supplementary Table S3 for raw values). The direction of the effect is displayed (< denotes an increase from negative to positive valence; > denotes a decrease).

Parameters	Call Type	Std. Error	*z* Value	*Pr (>|z|)*	Valence	Means	SD	Variation
**Without Body Movements**								
*AMrate*	Grunt	0.30	−2.41	0.128	Neg	0.36	1.16	ns
					Pos	−0.27	1.04	
	Scream	1.14	−3.31	0.009	Neg	0.15	1.99	>
					Pos	−3.29	0.79	
	Squeal	0.84	−0.55	0.993	Neg	0.01	1.01	ns
					Pos	−0.09	1.83	
*F1mean*	Grunt	21.66	−2.71	0.028	Neg	10.91	63.95	>
					Pos	−6.94	82.25	
	Scream	87.92	11.48	<0.001	Neg	−2.05	50.11	<
					Pos	44.05	75.07	
**With Body Movements**								
*F0mean*	Grunt	0.08	0.21	0.996	Neg	0.02	0.23	ns
					Pos	−0.02	0.17	
	Squeal	0.25	2.59	0.041	Neg	−0.02	0.27	<
					Pos	0.22	0.14	
*Harmonicity*	Grunt	0.17	1.78	0.262	Neg	−0.07	0.43	ns
					Pos	0.08	0.58	
	Squeal	0.49	−1.67	0.317	Neg	0.02	0.61	ns
					Pos	−0.28	0.43	
*F1mean*	Grunt	29.73	−3.48	0.002	Neg	8.25	56.76	>
					Pos	−11.09	79.72	
	Scream	82.98	14.13	<0.001	Neg	−1.51	48.91	<
					Pos	24.97	45.08	

## Supplementary Materials

The following are available online at http://www.mdpi.com/2076-2615/8/6/85/s1: Supplementary Methods. Acoustic analysis. Descriptions of the parameters measured and the analyses. Supplementary Tables: Table S1. Effect of emotional valence on vocal parameters (raw values; see Table 4 for statistics). Table S2. Effects of type of call on vocal parameters (raw values; see Table 6 for statistics). Table S3. Effects of interaction between valence and call type (raw values; see Table 7 for statistics). Audio S1. Wild boar grunt. Audio file of the grunt shown in Figure 1a. Audio S2. Wild boar scream. Audio file of the scream shown in Figure 1b. Audio S3. Wild boar squeal. Audio file of the squeal shown in Figure 1c.
