# Supplementary material for: Encoding of Emotional Valence in Wild Boar (Sus scrofa) Calls
Source: Animals (Basel). 2018 Jun 5;8(6):85. doi: 10.3390/ani8060085 (PMC6025020; doi:10.3390/ani8060085)
Supplement: Supplementary file 1 [file animals-08-00085-s001.zip › Maigrot et al. Supplementary material.docx]

Encoding of emotional valence in wild Boars (*Sus scrofa*) calls - Supplementary material

Supplementary Methods: Acoustic analysis

In this section, we provide a detailed description of the acoustic analysis. The methods we used for extracting source-related acoustic features, formants and intensity features (16 parameters; see Table 3 for a list and description) are detailed below (Praat commands are indicated in brackets). We extracted all vocal parameters using a custom built program in Praat, which batch processed the analyses and the exporting of output data [1].

Source-related vocal parameters were measured in grunts and squeals by extracting the fundamental frequency contour of each call. We extracted F0 contours using a cross-correlation method ([Sound: To Pitch (cc) command]; Grunts: time step = 0.01 s, pitch floor = 20-50 Hz, pitch ceiling = 50-100 Hz; Squeals: time step = 0.01 s, pitch floor = 150-250 Hz, pitch ceiling = 250-400 Hz). Using this method, we were able to follow F0 throughout all the calls. During the extraction of the pitch contour, octave jumps were rectified if necessary and incorrect values were “unvoiced” in the Pitch edit window, by comparing the pitch contour detected by the program [Inspect Pitch object] command to the F0 visible on a spectrogram edited with the following settings (FFT method, time steps = 1000, frequency steps = 250, Gaussian window shape, dynamic range = 60 dB, grunts: window length = 0.07-0.1 s, view range = 0-1000 Hz; squeals: window length = 0.1-0.18 s, view range = 0-8000 Hz; Fig. 1). F0 was not measured in screams, since these calls are not tonal and no F0 is visible. We also measured the harmonicity of all the calls (Harmonicity), which is the degree of acoustic periodicity calculated as the ratio between the signal itself and the background noise.

Additionally, we included in our analyses the frequency values at the upper limit of the first (Q25), second (Q50) and third (Q75) quartiles of energy, measured on a linear amplitude spectrum applied to the whole call. These values describe the relative energy distribution in the spectrum. We also included in our analyses the total duration of each call (Duration), measured directly on the spectrogram.

We then measured intensity characteristics by extracting the intensity contour of each call [Sound: To Intensity command]. We included in our analyses the mean variation per second (AMvar), calculated as the cumulative variation in amplitude divided by the total call duration; the number of complete cycles of amplitude modulation per second (AMrate) and the mean peak-to-peak variation of each amplitude modulation (AMextent) [2].

Finally, we measured filter-related (formants) acoustic features in grunts and screams by extracting the contour of the first three formants of each call using Linear Predictive Coding analysis (LPC; [Sound: To Formant (burg) command], time step = 0.1 s, maximum number of formants = 4–6, maximum formant = 1100–4000 Hz, window length = 0.2 s). We compared the outputs of the LPC analysis with visual inspections of spectrograms to check if Praat accurately tracked the formants. Spurious values were deleted and we corrected for octave jumps when necessary. We included the mean (F1-3mean) and the range (F1-3range) values of the formants in our analyses. Formants were not measured in squeals, which are tonal and high frequency sounds without strong frequency modulations. These features result in low spectral density and hence low formant resolution [3].

References

1. Reby, D.; McComb, K. Anatomical constraints generate honesty: acoustic cues to age and weight in the roars of red deer stags. Anim. Behav. 2003, 65, 519–530, doi:10.1006/anbe.2003.2078.

2. Charlton, B. D.; Zhihe, Z.; Snyder, R. J. Vocal cues to identity and relatedness in giant pandas (Ailuropoda melanoleuca). J. Acoust. Soc. Am. 2009, 126, 2721–2732, doi:10.1121/1.3224720.

3. Charlton, B. D.; Taylor, A. M.; Reby, D. Function and Evolution of Vibrato-like Frequency Modulation in Mammals. Curr. Biol. 2017, 27, 2692–2697.e3, doi:10.1016/j.cub.2017.07.046.

Supplementary Tables: Tables S1, S2 and S3 (Raw values for the vocal parameters)

Audio S1. Wild boars grunt. Audio file of the grunt shown in Figure 1 (a)

Audio S2. Wild boars scream. Audio file of the scream shown in Figure 1 (b)

Audio S3. Wild boars squeal. Audio file of the squealshown in Figure 1 (c)

**Table S1.** Effect of emotional valence on vocal parameters (raw values; see Table 4 for statistics).

| **Parameter** | **Negative** | |  | **Positive** | |
| --- | --- | --- | --- | --- | --- |
|  | **Mean** | **SD** |  | **Mean** | **SD** |
| F0mean | 144.68 | 117.03 |  | 65.07 | 46.99 |
| F0AbsSlope | 155.69 | 189.20 |  | 86.72 | 117.29 |
| AMextent | 6.02 | 3.72 |  | 5.43 | 4.50 |
| AMrate | 5.06 | 2.55 |  | 2.55 | 1.21 |
| Q25 | 248.23 | 219.88 |  | 105.94 | 59.48 |
| Q50 | 542.08 | 398.82 |  | 262.25 | 155.88 |
| Q75 | 1218.88 | 706.77 |  | 783.26 | 444.87 |
| Duration | 0.87 | 0.65 |  | 0.42 | 0.19 |
| Harmonicity | 2.59 | 2.27 |  | 4.50 | 3.18 |
| F1mean | 345.63 | 103.32 |  | 274.22 | 187.93 |
| F1range | 230.77 | 146.88 |  | 164.83 | 76.69 |
| F2mean | 925.23 | 232.06 |  | 799.66 | 274.27 |
| F2range | 261.93 | 128.36 |  | 192.05 | 99.92 |
| F3mean | 1470.26 | 297.24 |  | 1371.35 | 382.65 |
| F3range | 304.61 | 142.09 |  | 191.08 | 83.14 |

**Table S2.** Effect of the type of call on vocal parameters (raw values; see Table 6 for statistics).

| **Parameter** | **Grunt** | |  | **Scream** | |  | **Squeal** | |
| --- | --- | --- | --- | --- | --- | --- | --- | --- |
|  | **Mean** | **SD** |  | **Mean** | **SD** |  | **Mean** | **SD** |
| F0mean | 57.37 | 16.18 |  | - | - |  | 245.49 | 105.56 |
| F0AbsSlope | 66.55 | 43.19 |  | - | - |  | 237.57 | 212.87 |
| AMextent | 5.52 | 4.30 |  | 6.45 | 3.51 |  | 5.28 | 2.78 |
| Amrate | 2.82 | 1.31 |  | 7.66 | 2.48 |  | 4.35 | 1.36 |
| Q25 | 109.24 | 43.35 |  | 333.08 | 263.25 |  | 269.99 | 183.36 |
| Q50 | 266.90 | 112.94 |  | 691.08 | 442.07 |  | 569.61 | 331.60 |
| Q75 | 791.91 | 365.96 |  | 1342.76 | 731.79 |  | 1269.64 | 676.99 |
| Duration | 0.45 | 0.22 |  | 0.95 | 0.67 |  | 0.96 | 0.58 |
| Harmonicity | 3.81 | 2.93 |  | - | - |  | 3.06 | 2.98 |
| F1mean | 274.01 | 106.30 |  | 413.19 | 217.75 |  | - | - |
| F1range | 180.93 | 107.37 |  | 232.94 | 112.19 |  | - | - |
| F2mean | 841.57 | 269.54 |  | 902.63 | 217.07 |  | - | - |
| F2range | 216.85 | 113.42 |  | 254.23 | 116.09 |  | - | - |
| F3mean | 1413.36 | 367.47 |  | 1424.31 | 268.04 |  | - | - |
| F3range | 238.03 | 134.40 |  | 293.68 | 129.45 |  | - | - |

**Table S3.** Effect of the interaction between valence and call type (raw values; see Table 7 for statistics).

| **Parameter** | **Grunt** | | | | **Scream** | | | | **Squeal** | | | |
| --- | --- | --- | --- | --- | --- | --- | --- | --- | --- | --- | --- | --- |
|  | **Negative** | | **Positive** | | **Negative** | | **Positive** | | **Negative** | | **Positive** | |
|  | **Mean** | **SD** | **Mean** | **SD** | **Mean** | **SD** | **Mean** | **SD** | **Mean** | **SD** | **Mean** | **SD** |
| F0mean | 58.88 | 15.51 | 56.42 | 16.60 | - | - | - | - | 244.83 | 106.37 | 251.65 | 112.92 |
| F0AbsSlope | 67.09 | 48.35 | 66.20 | 39.86 | - | - | - | - | 221.42 | 197.82 | 386.88 | 318.86 |
| AMextent | 5.95 | 4.25 | 5.20 | 4.34 | 6.16 | 3.10 | 12.66 | 7.52 | 5.36 | 2.68 | 4.52 | 3.97 |
| AMrate | 3.32 | 1.40 | 2.44 | 1.10 | 7.82 | 2.42 | 4.34 | 1.03 | 4.41 | 1.31 | 3.81 | 1.87 |
| Q25 | 130.35 | 53.25 | 95.93 | 28.94 | 339.91 | 267.06 | 182.75 | 66.90 | 269.33 | 184.31 | 276.08 | 201.43 |
| Q50 | 316.30 | 135.83 | 235.76 | 82.42 | 705.67 | 446.65 | 370.17 | 24.16 | 557.59 | 319.25 | 680.79 | 474.12 |
| Q75 | 896.76 | 397.25 | 725.81 | 330.18 | 1365.50 | 737.84 | 842.59 | 406.77 | 1227.85 | 635.25 | 1656.19 | 1023.47 |
| Duration | 0.52 | 0.25 | 0.41 | 0.19 | 0.97 | 0.67 | 0.38 | 0.15 | 1.00 | 0.60 | 0.62 | 0.14 |
| Harmonicity | 2.57 | 1.94 | 4.57 | 3.18 | - | - | - | - | 2.99 | 2.68 | 3.69 | 3.65 |
| F1mean | 299.02 | 81.92 | 258.10 | 116.94 | 387.80 | 102.00 | 959.12 | 1012.65 | - | - | - | - |
| F1range | 203.93 | 140.41 | 166.29 | 77.00 | 238.61 | 110.80 | 110.94 | 83.88 | - | - | - | - |
| F2mean | 902.75 | 250.17 | 802.19 | 275.55 | 923.24 | 208.85 | 572.04 | 74.05 | - | - | - | - |
| F2range | 252.27 | 123.39 | 194.05 | 100.80 | 261.06 | 114.20 | 107.39 | 8.04 | - | - | - | - |
| F3mean | 1477.99 | 332.41 | 1372.69 | 384.15 | 1439.55 | 264.32 | 1096.82 | 48.03 | - | - | - | - |
| F3range | 314.08 | 164.01 | 190.18 | 82.17 | 300.38 | 128.08 | 149.62 | 70.36 | - | - | - | - |
